# Implementation of self-management support in cancer care and normalization into routine practice: a systematic scoping literature review protocol

**DOI:** 10.1186/s13643-019-0952-5

**Published:** 2019-01-31

**Authors:** Doris Howell, Alison Richardson, Carl May, Lynn Calman, Rouhi Fazelzad, Saeed Moradian, Claire Foster

**Affiliations:** 1 University Health Network (Princess Margaret Cancer Centre), 610 University Ave, Room 15-617, Toronto, Ontario M5G2M9 Canada; 2 Faculty of Health Sciences, University of Southampton and University Hospital Southampton NHS Foundation Trust, Southampton, Hampshire UK; 3 Faculty of Health Sciences, University of Southampton, Southampton, UK; 4 Faculty of Health Sciences, University of Southampton, Southampton, UK

**Keywords:** Scoping review protocol, Cancer, Self-management, Implementation, Self-management support

## Abstract

**Background:**

Cancer survivors face a myriad of biopsychosocial consequences due to cancer and treatment that may be potentially mitigated through enabling their self-management skills and behaviors for managing illness. Unfortunately, the cancer system lags in its systematic provision of self-management support (SMS) in routine care, and it is unclear what implementation approaches or strategies work to embed SMS in the cancer context to inform health policy and administrator decision-making.

**Methods/design:**

A comprehensive scoping review study of the literature will be conducted based on methods and steps identified by Arksey and O’Malley and experts in the field. Electronic searches will be conducted in multiple databases including CINAHL, CENTRAL, EMBASE, PsycINFO, MEDLINE, AMED, Cochrane Database of Systematic Reviews, Database of Abstracts of Reviews of Effects (DARE) (up to Issue 2, 2015), ISI Proceedings (Web of Science), PsychAbstracts, and Sociological Abstracts from January 1997 to November 5, 2018. Following the PRISMA-Extension for Scoping Reviews (PRISMA-ScR), two authors will independently screen all titles/abstracts to determine eligibility, data will be abstracted by one author and checked by a second author, and findings will be narratively summarized based on constructs of implementation in the Normalization Process Theory.

**Discussion:**

This will be the first scoping review study to synthesize knowledge of implementation of SMS in the cancer care context and the implementation approaches and strategies on embedding in care. This information will be critical to inform health policy and knowledge end users about the necessary changes in care to embed SMS in practices and to stimulate future research.

**Electronic supplementary material:**

The online version of this article (10.1186/s13643-019-0952-5) contains supplementary material, which is available to authorized users.

## Background

Globally, the burden of cancer continues to grow with 21.4 million new cancer cases, 13.2 million cancer deaths, and 28.7 million cancer survivors estimated for 2030 worldwide [1]. Cancer patients experience significant physical, psychosocial, and spiritual health consequences due to the disease and treatment that are undertreated in clinical care resulting in greater morbidity that is burdensome to patients and the healthcare system [2–11]. Ultimately, it is patients (and families) that shoulder responsibility for self-management of illness to reduce the impact of these consequences on functioning in daily life and for adopting specific behaviors that can facilitate health recovery during and after treatment and to minimize late effect risks [12, 13].

A large body of empirical evidence has shown that enabling effective self-management of illness in non-malignant chronic diseases (i.e., diabetes) results in better disease control, reduced symptom severity, and a better quality of life (QOL), as well as lower health care utilization and costs [14–16]. Similarly, evidence is emerging in cancer that self-management interventions (SMI) and/or self-management support (SMS) programs have similar beneficial effects on reducing severity of physical and emotional distress and improving quality of life outcomes [17–20]. Thus, greater emphasis is now focused on the provision of SMS in healthcare systems to enable and empower cancer survivors with the knowledge and skills to assume a central role in the management of their cancer and recovery of health [19, 21].

Unfortunately, cancer healthcare systems lag other chronic diseases in the provision of SMS in routine care, which may leave cancer patients vulnerable to becoming sicker and at risk of worse survival [12]. This is not surprising given that implementation of SMS requires “whole-system” change inclusive of (1) training and guidance of patients in self-management, (2) development of clinicians’ skills so they can support patients to do this, and (3) change in care processes to enable these approaches to become a core element of care [22–24]. While there are systematic reviews of implementation of SMS in typical chronic conditions, i.e., diabetes, asthma, chronic obstructive pulmonary disease, and arthritis, cancer is noticeably absent from these reviews [14, 16, 25, 26]. It cannot be assumed, given the complex, multifaceted and dynamic (fluctuating disease course) nature of cancer as a chronic illness [27], that implementation of SMS programs mirroring approaches used in typical chronic conditions are applicable in the cancer care context. Implementation is also a complex endeavor in the context of the rapid, episodic nature of cancer care, and simply adding greater expectations to existing practice systems is unlikely to be successful [28].

To promote self-management on a wider scale, we need to understand implementation of SMS in the context of cancer care and the approaches and strategies that contribute to integrating and embedding SMS in cancer care. Despite existing reviews of effectiveness [17–20], none of these reviews have focused on implementation of SMS in the context of cancer care. Thus, the overall goal of this systematic scoping literature review is to identify what is known about the implementation of SMS interventions and/or programs (extent, range, and nature of research evidence). The specific aims of the review are twofold: (1) to characterize the nature of SMS interventions and/or programs being implemented (self-management intervention/program components) and emphasis placed on core skills and behavior change and (2) to identify the core implementation approaches and strategies (implementation intervention) that have been used and the factors that enable or hinder the work of embedding SMS in care (what works for whom and in what circumstances).

This review will help to inform health care decision-makers, providers, and researchers about the state of knowledge regarding implementation of SMS in the context of cancer care and the research gaps that need to be addressed. Scoping reviews are considered essential to organize evidence for decision-support [29].

## Methods/design

The study protocol is summarized in the PRISMA-Extension for Scoping Reviews (PRISMA-ScR) Checklist [30] (Additional file 1). Scoping literature reviews are intended to be comprehensive and systematically identify the breadth and depth of a body of literature [31], rather than focus on a narrow and specific research question that is typical of a systematic review for meta-analysis [32]. To ensure methodological rigor, we draw upon the scoping review framework and five-stage approach specified by Arksey and O’Malley [33] and additional steps specified by Levac [34], recommendations for consistency in approach [35]. The additional recommendations include clarifying and linking the purpose and research question in stage 1, balancing feasibility with breadth and comprehensiveness of the scoping process in stage 2, using an iterative team approach to selecting studies in stage 3 and extracting data in stage 4, incorporating a numerical summary and qualitative thematic analysis for reporting results, and considering the implications of study findings to policy, practice, or research in stage 5[35]. A sixth stage of consultation, meeting with stakeholders for translating knowledge, is also recommended [34] but will not be incorporated in this phase of the work. We plan to conduct a mixed-review, which is defined as reviews that include quantitative, qualitative, and mixed-method data sources and a parallel narrative synthesis approach with separate analysis of quantitative and qualitative data to interpret study results and map patterns in the data [36]. Qualitative studies will only be included if they are linked to a primary quantitative or mixed-method study of implementation of SMS and further elaborate on the implementation approach or strategies used. The five stages of this scoping literature review to be followed are described below:

### Stage 1: Identification of the research questions

Based on the PICOH acronym (population, intervention, comparison, outcomes, health care context) [37], our overall broad scoping review question is as follows: What is known in cancer populations (P—population) about the approaches to implementation of SMS interventions and/or programs (I—intervention) compared to usual care or other interventions such as implementation of patient education (C—comparator) on changes in outcomes (O—outcomes, may include four levels of change in patient knowledge, skills and/or behaviors, or intermediary variables, i.e., self-efficacy, health care provider knowledge/skills, care delivery processes, or system arrangements or health policy) in the context of cancer care (H—healthcare context: community-based programs, hospitals, primary care practices, ambulatory or outpatient care). The specific review questions to be addressed are:

1. What core implementation components, implementation theories or frameworks, and specific implementation strategies have been used to facilitate uptake and embedding of SMS interventions and/or programs in routine cancer care (implementation intervention)?
2. What populations (cancer patients and/or family members, community or peer support services, family physician practices, health care organizations, health care professionals) have been targeted for implementation of SMS?
3. What are the core components, skills, or behaviors that have been emphasized in the SMS interventions and/or programs being implemented (SMS intervention)?
4. What are the enabling or inhibiting factors that influence uptake and embedding of SMS in routine care?
5. What implementation outcomes, such as care processes, and/or patient or provider intermediary (self-efficacy, behaviors) outcomes or survivor health outcomes (symptoms, quality of life, psychological distress, functioning) or system level outcomes were measured (health care use, costs)?

### Stage 2: Identification of studies

Implementation interventions are complex interventions [38] that are not always well defined in the literature and various definitions have been proposed. Implementation has been defined as “deliberate initiation of a specified set of activities or strategies designed to put into practice an activity or program aimed to produce a change in patient or provider behaviors or the environments (change in care process or organizational structures) in which they operate” [39]. According to this definition, implementation processes are purposeful and should be described in sufficient detail such that independent observers can detect the presence and strength of a “specific set of activities” related to implementation [40]. Desired outcomes are achieved only when effective programs are implemented well using a systematic and staged approach to implementation [39]. There is a need to identify both the implementation approach including its core components and implementation strategies (i.e., educational outreach, audit and feedback) used to facilitate implementation (implementation intervention) and the core components and skills or behaviors emphasized in the SMS intervention or program or practice being implemented (SMS intervention).

#### Implementation interventions

Implementation interventions may target change at one or more levels (e.g., patients, health care providers, teams, organizations, care processes, or delivery system) [39]. Multifaceted implementation strategies (e.g., facilitation, audit, and feedback) that are tailored to address local contextual barriers to change are more likely to improve professional practice [41, 42]. Based on the commonalities among successful implementation programs, core implementation components have been identified and include (1) staff selection, (2) preservice and in-service training, (3) ongoing coaching and consultation, (4) staff evaluation, (5) decision support data systems, (6) facilitative administrative support, and (7) systems interventions [39] as outlined in Table 1.

**Table 1:** Components of implementation interventions

Component	Definition
Staff selection	Includes academic qualifications or level of experience of staff or peers selected to carry out the program, methods for recruiting and selecting practitioners to carry out the program.
Pre-service and in-service training	Strategies used to enhance understanding background information and rationale for key practices (may include communication channels), training on the program components and opportunities to practice new skills and receive feedback.
Ongoing coaching and consultation	A coach provides information, advice, encouragement, and opportunities to practice and use skills specific to the program or innovation; coaching for behavior change may target the practitioner, supervisory, or administrative support levels.
Staff performance assessment	Evaluation to assess the use and outcomes of skills taught in the training, and reinforced and expanded in coaching processes. Includes performance feedback for skill development and other measures for gauging implementation fidelity, or other assessments to ensure competent delivery of core intervention components.
Decision-support data systems	Process and outcome data, organizational fidelity measures, or collection of any data to support decision-making and progress in the implementation of the core intervention components over time.
Facilitative administration	Provision of leadership to support the overall processes of implementation and keep staff focused on achieving intervention outcomes. May include new policies and procedures, change in care structures (model of care or care processes), and promoting the climate and cultural shift required to support the change
Systems intervention	Strategies to work with external systems to ensure the availability of the financial, organizational, and human resources required to support the work of the practitioners.

The implementation strategies that can be used to facilitate uptake of a program or innovation have also been defined and are identified in a taxonomy developed by the Cochrane Effective Practice and Organization of Care Group (EPOC) [43] such as audit and feedback, local opinion leaders or champions, educational meetings, or patient-mediated approaches tailored to the local context (Table 2).

**Table 2:** Implementation strategies

Strategy	Description
Audit and feedback	A summary of health workers’ performance over a specified period of time, given to them in written, electronic, or verbal format. May include recommendations for clinical action.
Local opinion leaders/champions	The identification and use of identifiable local opinion leader to promote or champion good clinical practice.
Patient-mediated interventions	The use of patients to change professional practice; could include the provision of patient-reported outcomes to practitioners.
Public release of performance data	Informing the public about healthcare providers by the release of performance data in written or electronic form.
Reminders	Manualized or computerized interventions that prompt health care workers to perform and action during a consultation with a patient, i.e., computer decision support systems.
Educational games	The use of games as an education strategy to improve standards of care.
Educational materials	Distribution of individuals, groups, or educational materials to support clinical care, i.e., any intervention in which knowledge is distributed.
Education meetings	Courses, workshops, conferences of other educational meetings.
Educational outreach visits or academic detailing	Personal visits by a trained person to health care workers in their own settings, to provide information with the aim of changing practice.
Routine patient-reported outcome measures	Routine administration and reporting of patient-reported outcome measures to providers and/or patients.
Managerial supervision	Routine supervision visits by health staff.
Decision support tools such as guidelines	Evidence-based guidance on appropriate health care for specific clinical circumstances, e.g., symptom triage protocols.
Local consensus processes	Formal or informal local consensus processes, for example agreeing to a clinical protocol to manage a patient group, or for adapting a guideline.
Continuous quality improvement	An iterative process to review and improve care that includes involvement of healthcare teams, analysis of a process or system, a structured process improvement method or problem-solving approach and use of data to analyze changes, i.e., Plan, Do, Study, Act Cycles
Inter-professional education	Continuing education for healthcare professionals that involves more than one profession in joint, interactive learning.
Tailored interventions	Interventions to change practice that are selected based on an assessment of systematic assessment of barriers to change.

#### SMS intervention and/or program

In the context of cancer care, a difference has been drawn between patient self-management activities and supported self-management. Numerous definitions of self-management are used in the literature and may vary by country. We adopted the Institute of Medicine definition of self-management as “Involving [the person with the chronic disease] engaging in activities that protect and promote health, monitoring and managing of symptoms and signs of illness, managing the impacts of illness on functioning, emotions and interpersonal relationships and adhering to treatment regimens” [44]. This definition has been extended for post-treatment cancer survivorship as “awareness and active participation by the person in their recovery, recuperation, and rehabilitation, to minimise the consequences of treatment, promote survival, health and well-being” [45]. Optimal self-management entails the ability to apply core skills such as problem-solving and use cognitive, behavioral, and emotional strategies in the continual self-regulation of health and to maintain a satisfactory quality of life [46]. Self-management has been distinguished from self-care, which more broadly delineates the healthy lifestyle behaviors or preventive strategies undertaken by individuals to promote or to maintain health [44]. Whereas self-management support (SMS) also labelled supported self-management has been defined as the “systematic provision of education and supportive interventions by health care staff to increase patients’ skills and confidence in managing their health problems, including regular assessment of progress and problems, goal setting, and problem-solving support” [47] and to support daily decision-making to improve health-related behaviors and clinical outcomes [48]. SMS can be viewed in two ways: as a portfolio of techniques and tools to help patients adopt behaviors [48] and as a fundamental transformation of the patient-caregiver relationship into a collaborative partnership [49]. SMS is complementary to patient education to improve knowledge of disease, but it also differs as it focuses on the patients’ own agenda, improving problem-solving skills, and building the patient’s confidence (self-efficacy) in using those skills in coping with the broad range of biopsychosocial consequences inherent in living with a chronic illness [50], and encourages patients to become active participants in their own care through goal setting and action planning [51]. Improvements in self-management and behavior change have been shown for SMS programs that include disease-specific information and education and collaboration between patient and clinicians, alongside behavior change strategies such as goal setting, action planning, and the provision of regular follow-up [50, 52]. A taxonomy of SMS program components has been identified that has been shown to have clinical utility in cancer survivorship SMS programs [53]. For this review, we have further adapted this taxonomy based on reviews of SMS program in cancer [20, 54, 55] and expertise of the scoping review team (Additional file 2). SMS interventions in cancer can be further classified based on (a) type, depending on whether the intervention is focused on *adjustment* (i.e., facilitating transition from an acute phase to survivorship or psychological problems) or on *problems* (e.g., side effects of treatment); (b) delivery, including technology-assisted interventions; and (c) techniques used, which may involve information provision, self-monitoring, goal setting, action plans, and positive feedback [54, 55].

### Stage 3: Study selection

#### Search criteria

Scoping studies are iterative; thus, proposed search terms may need to be refined as the extent of literature in the field becomes evident [33]. The literature search strategy will be initially developed for MEDLINE in consultation with an information specialist who has expertise in cancer reviews (RH). The MEDLINE strategy can be found in Additional file 3: Figure S1. An initial test of the search terms will be conducted in MEDLINE to refine the terms, and the search strategy will be modified for all other databases. A second information specialist at the University Health Network (Toronto, ON) has conducted a peer review of the search strategy using the Peer Review of Electronic Search Strategies (PRESS) checklist, which is common practice for scoping reviews [56, 57]. Specific Medical Subject Headings (MeSH) and free text terms for *self-management*, *self-efficacy*, *self-management support* and related terms, i.e., “self-care” or “educat” or “activate,” will be combined with terms for “cancer”. These terms will be combined with implementation study terms, for example “real world,” “routine clinical care,” primary care, “Phase IV,” “knowledge translation,” “adoption,” “integration,” “dissemination” and “implementation” using Boolean logic operators (and, or).

#### Databases

Standard bibliographic databases will be used as follows: MEDLINE, EMBASE, CINAHL PsycINFO, AMED, Cochrane Database of Systematic Reviews, Database of Abstracts of Reviews of Effects (DARE) (up to Issue 2, 2015), ISI Proceedings (Web of Science), PsychoAbstracts, and Sociological Abstracts from January 1997 to November 5, 2018, or from inception and/or closure of the database if occurring within this timeframe. This literature review start date was chosen as the first publication of a refined Chronic Care Model developed through consensus of experts was published, which stimulated self-management research in chronic diseases [58–60]. We will not include dissertations/theses or conference abstracts. Implementation intervention studies or evaluation of implementation in health care systems is not always published in the extant literature; thus, we will search specific web sites of organizations that are leading work on the implementation of SMS including the American and Canadian Cancer Society, Macmillan Cancer Support, Stanford Chronic Disease Management, Australian Cancer Society and Flinders University, and the Health Foundation in the United Kingdom and the National Cancer Survivorship Initiative in England and by asking experts in the field who are members of the Canadian Cancer Survivorship Research Consortium (CCSRC) for other key studies they are aware through an email broadcast to members (www.ccsrc.ca).

#### Study screening and selection

Titles, abstracts, and full papers will be independently reviewed by two members of the research team, the research coordinator and a methodologist (Howell) to identify eligible studies. A third member of the team will resolve disagreements between the two reviewers. We will follow the steps for study screening specified by Higgins and Deeks [61] including the following: (1) merge all references into the End Note reference management database and remove duplicates, (2) examine titles and abstracts for obvious irrelevant studies for exclusion, (3) review of full papers to identify eligible papers; (4) contact authors for additional details about the study as necessary to determine eligibility (three attempts will be made to contact study authors by email), and (5) make final decisions for study inclusion. Forward reference searching will be conducted through manual searches of reference lists until saturation (no new references are identified). References and literature sources will be included in the literature review if they meet eligibility criteria for inclusion of studies (inclusion/exclusion) identified in Table 3.

**Table 3:** Eligibility criteria based on PICOH acronym

Inclusion criteria	
Term	Definition
P—population	Implementation studies of SMS programs targeted to adult cancer populations (age 18 and over) at any stage of the cancer trajectory (treatment, post-treatment survivorship, palliative or end of life care).
I—intervention	Any implementation intervention study that focused on, or incorporated strategies to support self-management and delivered as part of routine clinical service or in a community agency or organization. SMS program targeting patients and/or providers and/or changes in the delivery system in the context of cancer care. Study design: Implementation studies that include a range of methodologies: population-level randomized controlled trials or cluster trials, quasi-experimental prospective studies, retrospective controlled studies, interrupted time series, controlled before and after studies, case-control, uncontrolled before and after studies, observational studies, qualitative studies of implementation processes or strategies or factors enabling or hindering implementation.
C—comparator	Any comparator such as usual care or other intervention if relevant.
O—outcomes	Outcomes of interest are not restricted but could include (self-management/self-care behaviors/healthy lifestyle behaviors, symptoms, emotional distress or adjustment (depression/anxiety), quality of life, patient experience, self-efficacy/mastery, survival, empowerment, health care use and/or costs, biological markers of disease, and process or implementation outcomes (clinicians’ knowledge and skills, attendance at education sessions, change in care delivery processes as per EPOC).
H—healthcare context	Any health care setting that provides care to cancer populations; hospital, ambulatory or outpatient care, community services or organizations, primary care practices, remote (telehealth or other web-based designs).
Exclusion criteria Non-empirical sources, i.e., opinion papers, book chapters, guidelines, or editorials. Efficacy trials of self-management interventions in cancer that are not focused on implementation. Self-management interventions and/or programs that do not include at a minimum guided support to patients in the development of core skills and/or strategies to support change in behaviors to manage problems or adjust to cancer; or focus only on management of comorbidities. Patient education programs or interventions that do not emphasize patient acquisition of skills for self-management. Papers or studies not published in English.	

### Stage 4: Data extraction and charting the data

Charting describes a technique for synthesizing and interpreting data by sifting, charting, and sorting material based on the key issues, themes, and types of studies [33]. Reference manager software (End-Note) will be used to track citations and record each database searched, the years it covered, and search date. The research coordinator as the primary reviewer will extract data on all studies using a standard form and checked by a second reviewer. Data will be abstracted for each study using a data abstraction form to include (1) author(s), (2) year of publication, (3) source origin/country of origin, (4) aims/purpose, (5) study population (age, type of cancer, stage of disease/severity, phase in the cancer continuum, comorbidities, problem targeted, i.e., breathlessness or adjustment) and sample size (if applicable), (6) methodology or study type (qualitative studies will be further classified based on methodology and theoretical orientation if possible, i.e., grounded theory, ethnography, phenomenology), (7) intervention type (including mode) and comparator if applicable, (8) concept, (9) duration of the intervention (if applicable), (10) outcomes and measures used and any data on psychometric properties, and (11) key findings that relate to the review question. We will also extract specific detail about the SMS intervention and/or program on core components including (1) intervention target (HCPs, patients, carers, combinations, care process, or model of care delivery); (2) setting; (3) mode of delivery (group, individual, professional, lay led, joint led, face to face, telehealth) and group allocation (if applicable); (4) components (education, action plans, other techniques to support behavior change (tele)monitoring, support materials (written/electronic information) as per the EPOC taxonomy (Table 3) and the SMS taxonomy (Additional file 2). (5) duration and intensity of components; (6) follow-up (frequency and mode); (7) service arrangements (usual, primary/additional care, dedicated service); and (8) any cost-effectiveness data. The level of agreement between the two primary reviewers will be conducted on a training set of three studies and a level of agreement (*K*-statistic) calculated and modifications made if necessary prior to further abstraction. Differences will be resolved by consensus and engagement of a third reviewer. Data will be abstracted from eligible studies and entered in an ACCESS database. Qualitative studies will be uploaded into an NVIVO qualitative database [62].

### Stage 5. Collating, summarizing, and reporting the results

We will follow the PRISMA-Extension for Scoping Reviews (PRISMA-ScR) guidelines for the review and use a chart for recording the flow of data through the stages of the study selection process based on these guidelines [63]. Scoping studies seek to present an overview of all material reviewed to provide a narrative or descriptive account of findings [33]. However, controversy still exists as to whether the quality assessment of studies in scoping reviews is required [34]. Given the complexity of implementation studies and anticipated heterogeneity in implementation components and study designs, we will not appraise the quality of studies included.

### Data analysis and narrative synthesis approach

There is a lack of consensus regarding the best approach for synthesizing qualitative study findings [64] and in scoping literature reviews [65]. While many potential strategies are identified, narrative synthesis is common and remains a recommended approach [66]. Narrative synthesis is defined as an approach to the synthesis of evidence relevant to a wide range of questions that relies primarily on the use of words and text to summarize and explain—to “tell the story”—of the findings of multiple studies [67]. We will use strategies recommended in a narrative synthesis guidance document for summarizing and identifying patterns across studies including textual descriptions, tabulation, group and clustering, thematic analysis, and conceptual triangulation using concept mapping [66]. A textual summary of data from each study will be tabulated with separate tables for qualitative studies, mixed-method studies, and quantitative study types (observational versus interventions). Study types will be tabulated using descriptive statistics (i.e., frequencies and percentages) and intervention studies grouped by implementation target (i.e., patient or provider or change in care delivery system). First, we will abstract data from studies on implementation approach and components, implementation or knowledge translation theory or framework, implementation strategies used, population(s) targeted for implementation (implementation intervention), and components of the SMS intervention (SMS intervention) using taxonomic criteria (Table 1, Additional file 2). Second, we will treat abstracted data from quantitative studies as qualitative data and combine this with qualitative study for narrative synthesis. The abstracted data will be analyzed using directed content analysis methods to identify themes or patterns in the data and to characterize the enabling or inhibiting factors that influence the uptake or embedding of SMS in routine care. A directed content analysis approach is distinct from traditional inductive content analysis and is guided by a more structured process that uses existing theory (concepts or constructs) or variables from prior research, also called a framework or variable oriented analysis, i.e., key concepts/constructs and variables, as initial coding categories [67, 68]. This approach avoids reinterpretation of qualitative data, which has been criticized, and ensures the development of patterns or themes grounded in the data. Thirdly, we will code data abstracted from these studies using a coding taxonomy inclusive of coding definitions agreed among all members of the research team. The coding taxonomy will be derived from variables in the implementation approach (Table 1), implementation strategies (Table 2), and the self-management taxonomy (Additional file 2), and Normalization Process Theory (NPT) [69] (Table 4). Given that our goal is to identify patterns in the implementation of SMS support, we will do initial coding based on the concepts in our taxonomies. This will be followed by a second level of coding to characterize the enabling or inhibiting factors that influence the embedding of SMS in routine care based on constructs in NPT [70]. NPT supports the characterization of the “work” of implementation and provides an orienting framework to identify the social processes influencing the embedding or normalization of innovations such as SMS in routine practice, and has been shown to be useful for conceptualizing the enabling or hindering factors for embedding of innovations in routine clinical care [71]. NPT sensitizes analytical thinking to four determinants of embedding innovations, i.e., normalizing, in clinical practice as defined in Table 4. NPT has been used to examine embedding of innovations in practice, and we will build on a previously developed coding taxonomy adapted to address the aims for this study [72].

**Table 4:** NPT analysis for evaluation of implementation of SMS in the cancer context

NPT construct	Definition	Questions to Consider
*Coherence*	Meaning and sense-making by participants Refers to the extent to which technology or health care practice makes sense to stakeholders for successful adoption.	1. *Is the SMS intervention easy to describe?* 2. *Is it clearly distinct from other interventions?* 3. *Does it have a clear purpose that end-users understand?* 4. *Expected benefits and are they valued?* 5. *Will it fit with overall goals of the organization?*
*Cognitive participation*	Concerns the commitment and collective engagement of stakeholders	1. *Do end users think SMS is a good idea?* 2. *End users willingness to invest time in SMS?*
*Collective action*	Refers to the relationships and the work required for a new intervention to be taken up in practice and to identify the factors that serve as barriers to implementation and embedding	1. *Perceived impact on workload?* 2. *Promote or impede their work?* 3. *Compatible with existing work practices?* 4. *Impact on division of labor, resources or responsibility?* 5. *Fit with overall goals and activities of the organization?*
*Reflexive monitoring*	Participants reflect on or appraise the trial Successful embedding of resources and technologies in everyday practice relies upon a continuous process of evaluation that can feed back into refining the object of implementation to ensure it is fit for purpose.	1. *How do end-users perceive the intervention in use?* 2. *Perceived as advantageous to patients?* 3. *Ongoing monitoring of intervention uptake? Or adapting to local context?*

### Anticipated problems and mitigation strategies

Scoping reviews by their very nature seek to present an overview of a large and diverse body of evidence, and our yield of studies may be larger than expected. We will add specific filters to limit the yield if necessary without compromising the intent of the review, i.e., limiting search terms for SMS and implementation (e.g., remove terms such as policy, quality control, innovation), and restrict the review to quantitative studies, SMS interventions for symptom management, and/or ambulatory cancer sectors only. Interchangeable use of terms, i.e., self-management or self-care, may be challenging, and subsequently, both terms will be included in the search strategy to ensure we capture studies that have used both terms. However, we will only select studies where the focus is on the implementation of SMS and the SMS intervention includes an emphasis on building patient skills and training of patients to use specific behaviors for managing the effects of cancer. Finally, identification of qualitative studies can be problematic, and we use recommended terms for identification of those specific to implementation of SMS.

## Discussion

While there are reviews of the implementation of SMS in routine care and significant efforts underway in the UK to integrate SMS in routine care, the emphasis has been on non-malignant chronic conditions [24] and we were unable to identify any scoping reviews of implementation studies of SMS in the context of cancer care. This scoping review will enable examination of the breadth of literature specific to cancer populations. We anticipate the results will be valuable for informing health care policy and knowledge end-users’ decisions regarding implementation strategies that could be applied in the cancer system to facilitate uptake of SMS and will identify the research gaps that need to be addressed. We will disseminate the findings of the review to academic audiences using traditional methods of publications in journals and presentations at international conferences targeting health service and implementation science researchers and oncology clinicians. Additionally, we will capitalize on the dual roles of many of our investigators to disseminate the study results with key cancer or health care organizations such as Macmillan Cancer Support and Cancer Care Ontario, through networks such as the CCSRC and Canadian Cancer Survivor (patient) networks, and professional organizations such as the International Psychosocial Oncology Society (IPOS) and the International Society of Nurses in Cancer Care (ISNCC). Data for the study will be held for up to 10 years at the University Health Network.

## Additional files

Additional file 1: PRISMA Extension for Scoping Reviews (PRISMA-ScR) (PDF 616 kb)

Additional file 2: SMS Taxonomy (PDF 45 kb)

Additional file 3: **Figure S1.** Medline Search Strategy (PDF 265 kb)

## Abbreviations

NPT: Normalization Process Theory
PICOH: Population, intervention, comparison, outcomes, health care context
QOL: Quality of life
SMS: Self-management support
