# Supplementary material for: Implementation of self-management support in cancer care and normalization into routine practice: a systematic scoping literature review protocol
Source: Syst Rev. 2019 Jan 31;8:37. doi: 10.1186/s13643-019-0952-5 (PMC6354326; doi:10.1186/s13643-019-0952-5)
Supplement: Supplementary file 2 — SMS Taxonomy (PDF 45 kb) [file 13643_2019_952_MOESM2_ESM.pdf]

Supplement 1: Taxonomy of Self-Management Support: Adapted for Cancer Populations in the Context of Cancer Care

**Overarching Dimension**<br>Modes of Delivery (e.g. face-to-face, one-to-one, group, technology assisted (remote, tele-healthcare, web-based, telephone)<br>Personnel Delivering the Support (e.g. health care professional, peers, other i.e. psychology helper, co-tutor approach)<br>Targeting (e.g. individual, groups, service delivery system, generic or cancer specific, cultural or gender groups)<br>Intensity, frequency, duration of the Intervention (not the individual components)<br>Type: Adjustment-focused (e.g. facilitating transitions) or problem-focused (enhancing coping skills for specific problems or symptoms) or both.<br>Direct (to patients and families) or Indirect (enabling or training of health care professionals to deliver these components.<br>Intervention Approach: Goal setting; action plans; problem solving; self-monitoring; stress management; information provision; sharing experiences; counselling; coaching; motivational interviewing; positive feedback; peer modelling.		
**Component**	**Description**	**Examples**
1. Information about condition and/its management	Information and instruction about cancer, treatment toxicities and side effects and impact on body systems (physiology) and skills for self-monitoring, symptom recognition and specific management strategies, medication use for disease and/or symptom control.	Explanation of the effects of cancer treatment on body systems and pathophysiology of cancer.<br>Preparatory or anticipatory education prior to cancer treatment. Specific exercises for managing lymphedema due to lymph node dissection.
2. Information about managing psychosocial consequences of illness.	Information and instruction about the effects of cancer on emotions and social consequences of cancer and managing these to reduce impact in daily life. Includes understanding and managing stress, fear of recurrence or disease progression, sexuality and intimacy, and learning to cope with these effects and the psychological impact of uncertainty. Specific instruction on recognition of depressive symptoms and prolonged anxiety and urgency for reporting suicidal thoughts.	Living with uncertainty in cancer programs.<br>Solution-focused counselling and coping skills training programs to foster uptake of behaviours.
3. Information about available social support resources and navigating transitions.	Information (written, verbal, visual) on issues such as financial benefits, transportation for treatment, sources of social or peer support, instruction on skills for navigating health care systems and care transitions.	Advice on obtaining financial assistance and peer support programs other practical support (transportation for treatment).
4. Provision of /agreement on goal setting and specific clinical action plans and/or urgent problems.	Written instructions or care plans prepared with/or by a healthcare professional to enable the person to manage the bio-psychosocial effects of cancer, tailored to the person, cancer type and treatment modality, or required surveillance for post-treatment survivors. Includes specifics on symptom monitoring and recognition of symptoms of deterioration and/or recurrence and what actions to take.	Written action plan to enable self-adjustment of medications in response to cancer treatment toxicities and side-effects/adherence to oral agents.<br>Specific advice on post-treatment surveillance and management of long term effects, including symptom and signs (recognition) of disease recurrence (i.e. Survivorship Care Plan).
5. Regular clinical review.	A regular, scheduled review of the person, their condition and self-management, conducted by a health care professional.	Regular clinical visits reviewing the person's condition and self-management.

6. Monitoring of condition with feedback.	Monitoring symptoms, behaviours or objective measures related to cancer. Data must be fed back to the patient. Interpretation, decision, and/or action is taken by the patients, but may be supported by the professional. Professionals may support self-management by reviewing monitored data and providing feedback to the patient.	Patient reported outcomes feedback to patient and/or clinician for monitoring symptoms. Review of Prostate Specific Antigen or blood counts (patient access to reports through patient portals). Daily self-care or symptom diaries.
7. Practical support with adherence (medication or behavioural).	Provision of practical help to improve a person's adherence to mediation or behaviour change activities.	Diary of medication use. Medication reminders. Motivational interviewing or pharmacy review.
8. Provision of equipment	Specific instruction on use of specialized equipment for self-management of the long-term condition.	Chemotherapy pumps; administration of G-CSF or hormone injections, Equipment for erectile dysfunction.
9. Provision of access to advice or support when needed	Provision of practical help to improve a person's adherence to medication or behaviour change activities.	Contact details of specialist nurse helpline for information or support or to advise in the event of clinical deterioration *(telephone triage lines); Self-referral for supported self-management.*
10. Training rehearsal to communicate with health care professionals	Teaching patients to develop communication skills/techniques to improve relationships, better communicate needs, and enhance shared decision making with healthcare professionals. Also supporting/mentoring people to practise the skills they have been taught.	- Strategies for communicating with health care providers, such as taking a tape recorder to doctors' visits and recording consultation<br>- Community Support Workers from minority ethnic/deprived groups who provide advocacy and support communication with healthcare professionals, attending an appointment with the person
11. Training rehearsal for life style adaptations and for everyday activities	Teaching skills that support everyday activities or practical skills such as managing specific strategies for self-management on daily basis.	Dietary changes to manage side-effects of cancer treatment.
12. Training rehearsal for psychological strategies	May include problem solving strategies, cognitive symptom management, relaxation techniques, re-framing, cognitive re-structuring, goal setting and action planning (performance feedback of the behaviour/outcome of the behaviour.	Relaxation (including guided imagery, diaphragmatic breathing, mindfulness), gratitude activity, goal setting activity (including action planning), solution focussed goal feedback and rewards. Peer modelling i.e. sharing experiences; counselling; coaching; motivational interviewing; positive feedback; peer modelling.
13. Social support	Facilitation of social support (practical and emotional support), where a person feels cared for and supported by others in a social network. May include peer support, peer mentoring, and group socializing.	Encourage patients to join peer support programs.
14. Lifestyle advice and support	Education on specific self-management strategies to reduce late effect risks based on type of cancer treatment. May also include	Supervised exercise or rehabilitation programs for instance to address increased risk of late effects i.e.

	general lifestyle advice and counselling support concerning diet, physical activity, tobacco and alcohol. Including:<br>• physical activity and pacing for fatigue management.<br>• sleeping better<br>• body changes, sexuality and intimacy<br>• physical activity for reducing late effect risks<br>• healthy nutrition	metabolic syndrome and/or heart disease due to particular chemotherapy agents.
