# Supplementary material for: Implementation of self-management support in cancer care and normalization into routine practice: a systematic scoping literature review protocol
Source: Syst Rev. 2019 Jan 31;8:37. doi: 10.1186/s13643-019-0952-5 (PMC6354326; doi:10.1186/s13643-019-0952-5)
Supplement: Supplementary file 3 — Figure S1. Medline Search Strategy (PDF 265 kb) [file 13643_2019_952_MOESM3_ESM.pdf]

COURAGE LIVES HERE

UHN Health Sciences Libraries

Literature Search Results

For: Dr. Doris Howell	Date Completed: Oct 3, 2018
	Tel:
Department: Supportive Care	Fax:

Attached is your search for:

Scoping Review: implementation studies of self-management support in the context of cancer care, limited to 1997 to current, and English; excluded conference abstracts.

The databases searched were:

Ovid MEDLINE(R) 1946 to November 5, 2018

SEARCH STRATEGY: See below

SEARCH RESULTS: See attached

Search Completed By: Original searches done by Junhui Zhang, updated searches done by Rouhi Fazelzad, Information Specialist, 416-946-6539, rouhi.fazelzad@uhn.ca

It is important that you are satisfied with your search results.
If you have any questions regarding this search, or if the results were not satisfactory, please do not hesitate in contacting me.

To request items not available in our library system, an INTERLIBRARY LOAN REQUEST FORM can be obtained from the library's circulation desk or through the Virtual Library. Any questions regarding our Document Delivery Service can be directed to Walter by telephone at 416-340-4121 or by email at Walter.Schmanda@uhn.ca.

For any other circulation inquiries:

Toronto General Hospital Library: (416) 340-3429
Toronto Western Hospital Library: (416) 603-5750
Toronto Rehab Library: (416) 597-3422, ext. 3050
Princess Margaret Library: (416) 946-4482

* Library staff make every effort to provide accurate and complete database search results. However, we assume no liability for information retrieved, its interpretation, applications or omissions.

Ovid MEDLINE(R) 1946 to November 5, 2018

#	Searches	Results	Type
1	Oncology Service, Hospital/	1378	Advanced
2	Cancer Care Facilities/	4850	Advanced
3	exp Medical Oncology/	19530	Advanced
4	Radiation Oncology/	3580	Advanced
5	Surgical Oncology/	171	Advanced
6	exp Neoplasms/	3087040	Advanced
7	Cancer Survivors/	966	Advanced
8	neoplas*.mp,kw.	2651809	Advanced
9	paraneoplas*.mp,kw.	11919	Advanced
10	cancer*.mp,kw.	1373395	Advanced
11	tumo?r*.mp,kw.	1753540	Advanced
12	onco*.mp,kw.	434132	Advanced
13	metast*.mp,kw.	451034	Advanced
14	malignan*.mp,kw.	467333	Advanced
15	aberrant crypt foci.mp,kw.	1414	Advanced
16	acanthoma*.mp,kw.	667	Advanced
17	adamantinom*.mp,kw.	934	Advanced
18	adenocarc*.mp,kw.	206607	Advanced
19	adenofibrom*.mp,kw.	2286	Advanced
20	adenolymphom*.mp,kw.	1685	Advanced
21	adenomat*.mp,kw.	20454	Advanced
22	adenomyo*.mp,kw.	3173	Advanced
23	adenosarcom*.mp,kw.	534	Advanced
24	adenosquam*.mp,kw.	3223	Advanced
25	ameloblastom*.mp,kw.	3976	Advanced

26	androblastom*.mp,kw.	323	Advanced
27	angiofibrom*.mp,kw.	2024	Advanced
28	angiokeratom*.mp,kw.	1050	Advanced
29	angiolipom*.mp,kw.	591	Advanced
30	angioma*.mp,kw.	12164	Advanced
31	angiomyolipom*.mp,kw.	3500	Advanced
32	angiomyom*.mp,kw.	550	Advanced
33	angiosarcom*.mp,kw.	5187	Advanced
34	apudoma*.mp,kw.	559	Advanced
35	arrhenoblastom*.mp,kw.	348	Advanced
36	astrocytom*.mp,kw.	19938	Advanced
37	blastom*.mp,kw.	10150	Advanced
38	Bowen*.mp,kw.	3085	Advanced
39	Brenner*.mp,kw.	1382	Advanced
40	Buschke-Lowenstein*.mp,kw.	242	Advanced
41	carcin*.mp,kw.	878408	Advanced
42	cementoma*.mp,kw.	479	Advanced
43	chemodectomas*.mp,kw.	294	Advanced
44	cholangiocarcin*.mp,kw.	10728	Advanced
45	chondroblastom*.mp,kw.	1268	Advanced
46	chondroma*.mp,kw.	6081	Advanced
47	chordoma*.mp,kw.	3864	Advanced
48	chondrosarcom*.mp,kw.	8582	Advanced
49	choriocarcin*.mp,kw.	8847	Advanced
50	craniopharyngioma*.mp,kw.	5021	Advanced
51	cystadenofibrom*.mp,kw.	205	Advanced
52	cystosarcom*.mp,kw.	610	Advanced
53	cytoma*.mp,kw.	319	Advanced

54	dermatofibrosarcom*.mp,kw.	1768	Advanced
55	desmoplas*.mp,kw.	3762	Advanced
56	dysgerminoma*.mp,kw.	6030	Advanced
57	DCIS.mp,kw.	3952	Advanced
58	DSRCT.mp,kw.	236	Advanced
59	ependymom*.mp,kw.	6275	Advanced
60	Ewing*.mp,kw.	9396	Advanced
61	fibroadenom*.mp,kw.	3917	Advanced
62	fibroepithelial*.mp,kw.	861	Advanced
63	fibroma*.mp,kw.	17794	Advanced
64	fibrosarcom*.mp,kw.	16080	Advanced
65	FAMMM.mp,kw.	70	Advanced
66	gangliogliom*.mp,kw.	1412	Advanced
67	ganglioneurom*.mp,kw.	3042	Advanced
68	gastrinoma*.mp,kw.	1790	Advanced
69	germinoma*.mp,kw.	3818	Advanced
70	glioblastom*.mp,kw.	31783	Advanced
71	glioma*.mp,kw.	52493	Advanced
72	gliosarcom*.mp,kw.	1148	Advanced
73	glomus jugulare*.mp,kw.	1325	Advanced
74	glomus tympanicum*.mp,kw.	249	Advanced
75	glucagonoma*.mp,kw.	1090	Advanced
76	gonadoblastom*.mp,kw.	766	Advanced
77	GCTOB.mp,kw.	6	Advanced
78	GIST?.mp,kw.	5759	Advanced
79	hemangioendotheliom*.mp,kw.	3997	Advanced
80	hemangiom*.mp,kw.	31377	Advanced
81	hemangiopericytom*.mp,kw.	3443	Advanced

82	hemangiosarcom*.mp,kw.	6987	Advanced
83	hamartoblastom*.mp,kw.	46	Advanced
84	hepatoblastom*.mp,kw.	3253	Advanced
85	hepatoma*.mp,kw.	26524	Advanced
86	histiocytom*.mp,kw.	8222	Advanced
87	hodgkin*.mp,kw.	83604	Advanced
88	nonhodgkin*.mp,kw.	113	Advanced
89	(hutchinson* adj2 freckle*).mp,kw.	642	Advanced
90	HNPCC.mp,kw.	2093	Advanced
91	immunocytom*.mp,kw.	593	Advanced
92	incidentaloma?.mp,kw.	1622	Advanced
93	insulinoma*.mp,kw.	6945	Advanced
94	kasabach merrit*.mp,kw.	654	Advanced
95	leiomyoblastom*.mp,kw.	392	Advanced
96	leiomyom*.mp,kw.	21840	Advanced
97	leiomyosarcom*.mp,kw.	10784	Advanced
98	leukem*.mp,kw.	286615	Advanced
99	preleukem*.mp,kw.	2006	Advanced
100	leukoplak*.mp,kw.	5991	Advanced
101	li-fraumeni*.mp,kw.	1142	Advanced
102	lipoblastom*.mp,kw.	431	Advanced
103	lipoma*.mp,kw.	17104	Advanced
104	liposarcom*.mp,kw.	6248	Advanced
105	luteoma*.mp,kw.	213	Advanced
106	lymphangio*.mp,kw.	13663	Advanced
107	lymphoblastom*.mp,kw.	360	Advanced
108	lymphocytom*.mp,kw.	324	Advanced
109	lymphoma*.mp,kw.	211907	Advanced

110	lymphosarcom*.mp,kw.	5059	Advanced
111	lynch*.mp,kw.	3029	Advanced
112	macroglobulinem*.mp,kw.	5707	Advanced
113	m?croprolactinom*.mp,kw.	708	Advanced
114	mastocytom*.mp,kw.	1989	Advanced
115	mastocytos?s*.mp,kw.	3676	Advanced
116	medulloblastom*.mp,kw.	8505	Advanced
117	meigs*.mp,kw.	836	Advanced
118	melanoameloblastom*.mp,kw.	11	Advanced
119	melanoblastom*.mp,kw.	489	Advanced
120	melanocarcin*.mp,kw.	100	Advanced
121	melanoma*.mp,kw.	111605	Advanced
122	melanosis.mp,kw.	3965	Advanced
123	melanotic*.mp,kw.	2829	Advanced
124	meningiom*.mp,kw.	21844	Advanced
125	mesenchymom*.mp,kw.	2040	Advanced
126	mesoblast*.mp,kw.	722	Advanced
127	mesonephrom*.mp,kw.	1161	Advanced
128	mesotheliom*.mp,kw.	15927	Advanced
129	metaplas*.mp,kw.	21863	Advanced
130	micrometast*.mp,kw.	5898	Advanced
131	muir-torre*.mp,kw.	385	Advanced
132	myelolipom*.mp,kw.	966	Advanced
133	myoepitheliom*.mp,kw.	1233	Advanced
134	myofibrom*.mp,kw.	708	Advanced
135	myeloma*.mp,kw.	52544	Advanced
136	myoma*.mp,kw.	5633	Advanced
137	myosarcom*.mp,kw.	401	Advanced

138	myxofibrosarcom*.mp,kw.	382	Advanced
139	myxoma*.mp,kw.	9327	Advanced
140	myxosarcom*.mp,kw.	453	Advanced
141	n?evocarcin*.mp,kw.	78	Advanced
142	neurilemmom*.mp,kw.	13257	Advanced
143	neurocytom*.mp,kw.	752	Advanced
144	neuroectodermal*.mp,kw.	7747	Advanced
145	neurofibroma*.mp,kw.	18908	Advanced
146	neurofibrosarcom*.mp,kw.	465	Advanced
147	neurilemmom*.mp,kw.	13257	Advanced
148	neuroblastom*.mp,kw.	37418	Advanced
149	neuroma*.mp,kw.	13178	Advanced
150	neurothekeom*.mp,kw.	243	Advanced
151	NSCLC.mp,kw.	27996	Advanced
152	odontoma*.mp,kw.	1378	Advanced
153	oligodendrogliom*.mp,kw.	4996	Advanced
154	oligometast*.mp,kw.	806	Advanced
155	osteoblastom*.mp,kw.	1127	Advanced
156	osteochondrom*.mp,kw.	2880	Advanced
157	osteoclastom*.mp,kw.	324	Advanced
158	osteoma*.mp,kw.	12856	Advanced
159	osteosarcom*.mp,kw.	26069	Advanced
160	papilloma*.mp,kw.	60375	Advanced
161	papillary*.mp,kw.	57997	Advanced
162	paragangliom*.mp,kw.	7616	Advanced
163	pheochromocytom*.mp,kw.	20296	Advanced
164	phyllo?des*.mp,kw.	2118	Advanced
165	pinealocytoma*.mp,kw.	14	Advanced

166	pinealoma*.mp,kw.	1849	Advanced
167	pineoblastoma*.mp,kw.	348	Advanced
168	pineocytoma*.mp,kw.	240	Advanced
169	plasmacytom*.mp,kw.	10099	Advanced
170	(polycythem* adj2 vera?).mp,kw.	7113	Advanced
171	prolactinom*.mp,kw.	3842	Advanced
172	retinoblastom*.mp,kw.	20321	Advanced
173	rhabdoid*.mp,kw.	2209	Advanced
174	rhabdomyom*.mp,kw.	1604	Advanced
175	rhabdomyosarcom*.mp,kw.	13143	Advanced
176	sarcom*.mp,kw.	117035	Advanced
177	seminoma*.mp,kw.	7012	Advanced
178	Sertoli- Leydig.mp,kw.	1102	Advanced
179	somatostatinoma*.mp,kw.	436	Advanced
180	somatotrophinom*.mp,kw.	71	Advanced
181	struma ovarii*.mp,kw.	601	Advanced
182	thecoma*.mp,kw.	1031	Advanced
183	teratocarcin*.mp,kw.	2777	Advanced
184	teratoma*.mp,kw.	20372	Advanced
185	thymom*.mp,kw.	10041	Advanced
186	trophoblast*.mp,kw.	23078	Advanced
187	vipoma*.mp,kw.	588	Advanced
188	wilms*.mp,kw.	11820	Advanced
189	or/1-188	4093224	Advanced
190	Self-Management/	697	Advanced
191	exp Self Care/	50444	Advanced
192	Self Administration/	10651	Advanced
193	Self Medication/	4476	Advanced

194	Self Efficacy/	17599	Advanced
195	Patient Participation/	22945	Advanced
196	exp Cognitive Therapy/	24260	Advanced
197	self car*.mp,kw.	37538	Advanced
198	selfcar*.mp,kw.	93	Advanced
199	self manag*.mp,kw.	13178	Advanced
200	selfmanag*.mp,kw.	27	Advanced
201	self administ*.mp,kw.	38997	Advanced
202	selfadminist*.mp,kw.	46	Advanced
203	self medicat*.mp,kw.	6426	Advanced
204	selfmedicat*.mp,kw.	12	Advanced
205	self monitor*.mp,kw.	10039	Advanced
206	selfmonitor*.mp,kw.	15	Advanced
207	self efficac*.mp,kw.	26299	Advanced
208	selfefficac*.mp,kw.	17	Advanced
209	self guid*.mp,kw.	399	Advanced
210	selfguid*.mp,kw.	0	Advanced
211	self regulat*.mp,kw.	8505	Advanced
212	selfregulat*.mp,kw.	24	Advanced
213	self direct*.mp,kw.	4072	Advanced
214	selfdirect*.mp,kw.	9	Advanced
215	self determin*.mp,kw.	3912	Advanced
216	selfdetermin*.mp,kw.	3	Advanced
217	(personal adj2 manag*).mp,kw.	598	Advanced
218	((patient? or client?) adj2 participat*).mp,kw.	36954	Advanced
219	[((patient? or client?) adj2 empower*).mp,kw.]	0	Advanced
220	[((patient? or client?) adj2 activation*).mp,kw.]	0	Advanced
221	[((patient? or client?) adj2 engag*).mp,kw.]	0	Advanced

222	(cognitive adj3 therap*).mp,kw.	27400	Advanced
223	(psychoeducat* or psycho-educat*).mp,kw.	4529	Advanced
224	(nurs*-led adj6 surviv*).mp,kw.	15	Advanced
225	(peer-led adj6 surviv*).mp,kw.	2	Advanced
226	or/190-225	196454	Advanced
227	Health Plan Implementation/	5178	Advanced
228	Regional Health Planning/	5281	Advanced
229	Health Planning Guidelines/	4008	Advanced
230	Health Care Reform/	31451	Advanced
231	Organizational Policy/	13665	Advanced
232	Policy/	1905	Advanced
233	Policy Making/	15071	Advanced
234	Decision Making/	85198	Advanced
235	Organizational Innovation/	23361	Advanced
236	"Delivery of Health Care, Integrated"/	11176	Advanced
237	Program Development/	27021	Advanced
238	"Diffusion of Innovation"/	16626	Advanced
239	Information Dissemination/	14646	Advanced
240	Translational Medical Research/	8695	Advanced
241	Pragmatic Clinical Trials As Topic/	209	Advanced
242	Quality Improvement/	18007	Advanced
243	implement*.tw,kw.	322076	Advanced
244	(plan* adj2 manag*).mp,kw.	11222	Advanced
245	(plan* adj2 guideline?).mp,kw.	4497	Advanced
246	(care? adj2 reform*).mp,kw.	34291	Advanced
247	(policy or policies).tw,kw.	183533	Advanced
248	(decision? adj2 (maker? or making)).tw,kw.	104654	Advanced
249	innovat*.tw,kw.	86820	Advanced

250	diffusion.tw,kw.	131599	Advanced
251	disseminat*.tw,kw.	98535	Advanced
252	((knowledge or kt) adj2 translat*).mp,kw.	2838	Advanced
253	((knowledge or kt) adj2 transfer*).mp,kw.	1920	Advanced
254	((knowledge or kt) adj2 manag*).mp,kw.	2567	Advanced
255	((knowledge or kt) adj2 exchang*).mp,kw.	709	Advanced
256	((knowledge or kt) adj2 mobilit*).mp,kw.	24	Advanced
257	((knowledge or kt) adj2 diffus*).mp,kw.	174	Advanced
258	((knowledge or kt) adj2 creat*).mp,kw.	821	Advanced
259	((knowledge or kt) adj2 utili*).mp,kw.	813	Advanced
260	(knowledge adj2 care?).mp,kw.	2233	Advanced
261	(program* adj2 develop*).mp,kw.	55615	Advanced
262	(program* adj2 (improv* or outcome?)).mp,kw.	17256	Advanced
263	(organi?ational adj2 chang*).mp,kw.	2819	Advanced
264	(behavio?r* adj2 chang*).mp,kw.	35761	Advanced
265	(pragmatic adj2 trial?).mp,kw.	2050	Advanced
266	(qualit* adj2 improv*).mp,kw.	98972	Advanced
267	(framework? or frame work?).mp,kw.	165447	Advanced
268	adoption*.tw,kw.	34264	Advanced
269	enabling.mp,kw.	49697	Advanced
270	"real world".mp,kw.	20220	Advanced
271	"plan-do-study-act".mp,kw.	411	Advanced
272	"participatory action research".mp,kw.	840	Advanced
273	StaRI.mp,kw.	258	Advanced
274	PDSA.mp,kw.	233	Advanced
275	Implementation Science*.jw.	1056	Advanced
276	or/227-275	1351901	Advanced
277	189 and 226 and 276	4356	Advanced

278	limit 277 to "all child (0 to 18 years)"	481	Advanced
279	limit 277 to "all adult (19 plus years)"	2723	Advanced
280	278 not 279	120	Advanced
281	277 not 280	4236	Advanced
282	limit 281 to (clinical conference or consensus development conference or consensus development conference, nih or news or newspaper article)	27	Advanced
283	281 not 282	4209	Advanced
284	limit 283 to yr="1997 -Current"	3903	Advanced
285	limit 284 to english language	3694	Advanced
286	aware*.mp,kw.	169900	Advanced
287	(uptake or up-take).mp,kw.	317377	Advanced
288	(takeup or take-up).mp,kw.	7961	Advanced
289	adhere*.mp,kw.	170823	Advanced
290	concordan*.mp,kw.	51122	Advanced
291	accordan*.mp,kw.	50755	Advanced
292	(comply* or complies or compliance).mp,kw.	145144	Advanced
293	spread*.mp,kw.	164080	Advanced
294	barrier?.mp,kw.	211473	Advanced
295	facilitat*.mp,kw.	388942	Advanced
296	advice?.mp,kw.	38203	Advanced
297	advise?.mp,kw.	25276	Advanced
298	standard?.mp,kw.	1357997	Advanced
299	statement?.mp,kw.	50576	Advanced
300	or/286-299	2839360	Advanced
301	189 and 226 and 300	5399	Advanced
302	limit 301 to "all child (0 to 18 years)"	696	Advanced
303	limit 301 to "all adult (19 plus years)"	3745	Advanced

304	302 not 303	141	Advanced
305	301 not 304	5258	Advanced
306	limit 305 to (clinical conference or consensus development conference or consensus development conference, nih or news or newspaper article)	23	Advanced
307	305 not 306	5235	Advanced
308	limit 307 to yr="1997 -Current"	4842	Advanced
309	limit 308 to english language	4625	Advanced
310	285 or 309	6685	Advanced
311	310 not 285	2991	Advanced
312	empower*.mp,kw.	17459	Advanced
313	activat*.mp,kw.	1495996	Advanced
314	engag*.mp,kw.	119413	Advanced
315	integrat*.mp,kw.	367587	Advanced
316	312 or 313 or 314 or 315	1946537	Advanced
317	189 and 226 and 316	1679	Advanced
318	limit 317 to (english language and yr="1997 - Current")	1538	Advanced
319	285 or 318	4623	Advanced
